# Two serial MoCA assessments may support biomarker-sparing triage between Parkinson’s disease/Lewy body dementia and frontotemporal dementia: a progressive inclusion analysis of 1,129 participants

**DOI:** 10.3389/fnagi.2026.1848105

**Published:** 2026-08-26

**Authors:** Wei Lin, Sanjeet S. Grewal, Richard W. Byrne

**Affiliations:** Department of Neurologic Surgery, Mayo Clinic, Jacksonville, FL, United States

**Keywords:** aging, biomarker-sparing triage, cognitive trajectory, frontotemporal dementia, machine learning, Montreal Cognitive Assessment, Parkinson’s disease, serial assessment

## Abstract

Reliable differentiation of Parkinson’s disease/Lewy body dementia (PD/LBD) from frontotemporal dementia (FTD) affects treatment strategy and clinical trial eligibility, yet confirmatory biomarker testing remains costly and unevenly available. Whether routine cognitive trajectories can support testing prioritization has not been systematically quantified. We performed a progressive inclusion analysis of 1,129 National Alzheimer’s Coordinating Center participants with PD/LBD (*n* = 385) or FTD (*n* = 744) to determine the minimum number of serial Montreal Cognitive Assessment (MoCA) administrations required for diagnostic separation. Random forest classifiers used seven MoCA subdomain slopes from the first k chronological assessments (*k* = 2 through *k* = 8), with five-fold stratified cross-validation and 500-iteration bootstrap confidence intervals. In the primary full-cohort analysis, two assessments yielded AUC = 0.785 (95% CI 0.757–0.814; sensitivity = 0.922; specificity = 0.525). The interval-restricted 6–12 month subset yielded AUC = 0.837 (95% CI 0.787–0.885), near the lower edge of published biomarker-panel ranges in an indirect comparison, although its lower confidence bound remained below 0.85. Discrimination persisted after age matching (AUC = 0.794, 95% CI 0.761–0.826; residual age gap = −0.3 years) and age restriction to 55–75 years (AUC = 0.777, 95% CI 0.741–0.814). Age alone yielded lower discrimination (AUC = 0.721, 95% CI 0.691–0.751) than MoCA slopes, while slopes plus age yielded AUC = 0.867 (95% CI 0.845–0.888). Four-assessment performance was AUC = 0.831 (95% CI 0.791–0.873), consistent with an apparent sample-size-limited plateau as eligible N contracted from 391 at *k* = 4 to 98 at *k* = 6. These findings support serial MoCA trajectory analysis as an exploratory tool for prioritizing confirmatory diagnostic testing, with age sensitivity, subtype sensitivity, and operating-point performance quantified.

## Introduction

Differentiating Parkinson’s disease and Lewy body dementia (PD/LBD) from frontotemporal dementia (FTD) is a persistent clinical challenge that shapes treatment decisions, prognostic counseling, and eligibility for disease-modifying trials (McKeith et al., 2017; Rascovsky et al., 2011). The two disease groups share overlapping behavioral and cognitive features – executive dysfunction, apathy, and visuospatial impairment appear in both – yet their underlying neuropathology, pharmacological management, and disease trajectories diverge substantially (Gorno-Tempini et al., 2011; McKeith et al., 2017, 2020; Postuma et al., 2015; Rascovsky et al., 2011). Cholinesterase inhibitors benefit PD/LBD but lack evidence in behavioral variant FTD; dopaminergic therapy targets motor symptoms of PD/LBD but is irrelevant to most FTD presentations; and behavioral management strategies differ between the disinhibition and compulsive behaviors of FTD and the visual hallucinations and fluctuating cognition of Lewy body disease. Misclassification therefore delays appropriate intervention and may expose patients to ineffective or harmful therapies.

The past decade has seen rapid advances in fluid and imaging biomarkers for neurodegenerative disease. Cerebrospinal fluid (CSF) neurofilament light chain, amyloid-beta/tau ratios, and alpha-synuclein seed amplification assays now achieve AUC values of 0.85–0.95 for distinguishing synucleinopathies from tauopathies in specialized research cohorts (Hansson et al., 2022; Siderowf et al., 2023). Plasma phosphorylated tau and glial fibrillary acidic protein panels have shown similar promise (Ashton et al., 2022). These advances, however, carry practical constraints that limit their population-level impact. CSF collection requires lumbar puncture, a procedure that many older patients decline. PET imaging costs exceed $3,000 per scan in many health systems. Plasma biomarker panels, while less invasive, remain confined to reference laboratories in high-income countries and lack standardized cutoffs across assay platforms (Cummings et al., 2023). The World Health Organization estimates that over 55 million people live with dementia globally, with the majority in low- and middle-income countries where advanced biomarker infrastructure is largely absent (World Health Organization, 2021). A biomarker-sparing triage tool built on routinely collected clinical data could help prioritize confirmatory testing by identifying patients whose clinical assessment trajectories warrant faster biomarker or specialist evaluation.

The Montreal Cognitive Assessment (MoCA) is administered in over 200 countries and is already embedded in standard neurological follow-up protocols (Nasreddine et al., 2005). Its seven subdomain scores – attention, visuospatial/executive, orientation, language, delayed recall, abstraction, and naming – capture distinct cognitive dimensions that decline at different rates across neurodegenerative conditions. PD/LBD characteristically shows early visuospatial and attentional decline with relative preservation of language, while FTD, particularly the behavioral variant, presents with prominent executive and social cognition deficits and, in primary progressive aphasia variants, early language deterioration (Gorno-Tempini et al., 2011; Biundo et al., 2020; Rascovsky et al., 2011). Single-timepoint MoCA total scores have limited diagnostic specificity for differentiating neurodegenerative conditions, because different disease processes can produce similar aggregate scores through different subdomain combinations. Longitudinal subdomain trajectories, by contrast, encode disease-specific patterns of cognitive decline that cross-sectional snapshots cannot capture. Recent work has demonstrated machine learning classification of cognitive impairment and dementia subtypes from cross-sectional MoCA features, with PPA achieving the best subtype separation (Gourdeau et al., 2026). Jiang and colleagues conducted a systematic review and meta-analysis of machine learning methods for cognitive impairment detection in Parkinson’s disease, reporting pooled AUC values above 0.80 across heterogeneous feature sets (Jiang et al., 2025). These studies establish that cognitive data carry substantial diagnostic information, but none has systematically determined the minimum number of serial MoCA assessments needed for clinically useful neurodegenerative disease triage.

Three questions remain unanswered. First, how many serial assessments constitute a minimum viable triage signal? A two-visit protocol, if sufficient, could be embedded in routine 6–12 month follow-up schedules already standard in movement disorder and memory clinics. Second, which cognitive domains drive earliest diagnostic separation, and does the feature hierarchy shift as trajectories lengthen? Third, at the individual patient level, how long until the classifier reaches a high predicted-probability threshold – a metric directly relevant to prioritizing escalation to biomarker testing? We addressed these questions through a progressive inclusion analysis of 1,129 NACC participants with PD/LBD or FTD, restricting each participant to their first k chronological MoCA assessments (*k* = 2 through *k* = 8) and evaluating classification performance, feature importance evolution, interval requirements, and patient-level time-to-predicted-probability-threshold analysis.

## Materials and methods

### Cohort

We analyzed de-identified National Alzheimer’s Coordinating Center (NACC) Uniform Data Set participants under Data Use Agreement #17206. Diagnoses were assigned by consensus clinical teams at participating Alzheimer’s Disease Research Centers using contemporaneous syndromic criteria captured in NACC etiologic diagnosis fields. PD diagnoses followed Movement Disorder Society clinical diagnostic criteria (Postuma et al., 2015), dementia with Lewy bodies followed consensus Lewy body criteria (McKeith et al., 2017, 2020), behavioral variant FTD followed Rascovsky criteria (Rascovsky et al., 2011), and primary progressive aphasia followed Gorno-Tempini criteria (Gorno-Tempini et al., 2011). The FTD group encompassed behavioral variant FTD, primary progressive aphasia, FTD with motor neuron disease, and other or unspecified FTD diagnoses, reflecting the clinical heterogeneity encountered in practice. Within the PD/LBD group, NACC etiologic flags indicated Lewy body disease in 231 participants and idiopathic Parkinson’s disease in 102 participants; because these fields are overlapping, 44 participants carried both flags and 96 carried neither definite flag. First-visit cognitive status in the PD/LBD group was normal in 73 participants, impaired-not-MCI in 22, MCI in 161, and dementia in 129. We therefore analyze PD/LBD as a combined Lewy-body-spectrum class rather than as mutually exclusive PD, Parkinson’s disease dementia, and dementia with Lewy bodies subgroups.

This study is reported in accordance with the TRIPOD statement and STROBE guidance for observational cohorts; where AI/ML-specific items apply, TRIPOD-AI guidance was followed. A related analysis by the same group examines full-length MoCA trajectories in an overlapping NACC sample; the present study addresses a distinct question, the minimum serial-assessment count for diagnostic testing prioritization, and does not duplicate those results. The two analyses address different questions (full-trajectory classification versus minimum-serial-assessment triage), and no result from the related analysis is used as evidence here.

Eligible participants carried PD/LBD or FTD diagnoses and had serial MoCA assessments suitable for slope estimation. The full eligible cohort included 1,163 participants; 34 participants whose first two visits were separated by less than 0.5 months were excluded from the *k* = 2 analysis, yielding 1,129 participants (385 PD/LBD, 744 FTD) for the minimum-data comparison. Baseline characteristics are summarized in Table 1.

**TABLE 1:** Baseline characteristics of the *k* = 2 analytic cohort.

Group	*N*	Age, mean (SD), y	Female, *n* (%)	Education, mean (SD), y	Baseline MoCA, mean (SD)
PD/LBD	385	70.6 (8.4)	91 (23.6)	16.5 (2.8)	22.6 (5.0)
FTD	744	58.4 (14.6)	351 (47.2)	15.8 (2.6)	22.7 (7.0)
Overall	1,129	62.5 (14.0)	442 (39.1)	16.0 (2.7)	22.7 (6.4)

Age and education are reported as mean (standard deviation). Percentages use the number of participants with non-missing sex as the denominator. Education values coded as NACC missing values were excluded from summary calculations.

Seven MoCA cognitive domains were scored as percent of maximum possible score: attention (6 points), visuospatial/executive (5 points), orientation (6 points), language (3 points), delayed recall (5 points), abstraction (2 points), and naming (3 points) (Nasreddine et al., 2005). For each domain, raw points were divided by the maximum possible points for that domain and multiplied by 100. Slopes were estimated from the first k chronological assessments for each participant. At *k* = 2, the slope was the difference in domain percent score divided by elapsed years between the first two visits. At *k* ≥ 3, ordinary least-squares slopes were estimated across available visits within the first k assessments when at least two finite domain scores were present.

### Progressive inclusion design

For each k in {2, 3, 4, 5, 6, 7, 8}, participants with at least k assessments contributed slope features estimated from their first k chronological visits. At *k* = 2, each domain slope was computed as the change in percent-of-maximum score divided by the inter-assessment interval in years (delta-per-year). At *k* ≥ 3, slopes were estimated with ordinary least squares regression of percent-of-maximum score on time. This progressive inclusion design allowed us to track how classification performance evolved as data accumulated, while the restriction to the first k visits ensured that each configuration reflected a realistic clinical scenario.

NACC missing, unknown, and not-assessed codes were treated as missing values. A participant contributed to a given k configuration only if all seven engineered domain slopes were finite after slope estimation. The 34 participants whose first two visits were separated by less than 0.5 months were excluded from the *k* = 2 analysis. No imputation was performed. Per-configuration attrition is reported in Table 2.

**TABLE 2:** Classification performance, subgroup, and sensitivity analyses.

Section	Configuration	N (PD/LBD)	N (FTD/Group 2)	N Total	Mean age PD/LBD	Mean age FTD	Residual age gap (y)	AUC	95% CI	Sensitivity	Specificity	Notes
A. Progressive inclusion	*k* = 2, all intervals	385	744	1,129	–	–	–	0.785	0.757–0.814	–	–	
A. Progressive inclusion	*k* = 3	385	371	756	–	–	–	0.812	0.777–0.843	–	–	
A. Progressive inclusion	*k* = 4 [threshold]	196	195	391	–	–	–	0.831	0.791–0.873	–	–	Apparent diminishing-returns threshold
A. Progressive inclusion	*k* = 5	77	103	180	–	–	–	0.856	0.789–0.914	–	–	Wide CI; overlaps *k* = 4
A. Progressive inclusion	*k* = 6	32	66	98	–	–	–	0.846	0.739–0.933	–	–	Wide CI; exploratory
B. Interval strata (*k* = 2)	6–12 months	137	94	231	–	–	–	0.837	0.787–0.885	–	–	Minimum viable assessment
B. Interval strata *(k* = 2)	12–18 months	192	471	663	–	–	–	0.837	0.800–0.872	–	–	
B. Interval strata (*k* = 2)	18+ months	56	176	232	–	–	–	0.731	0.654–0.803	–	–	Survivorship bias likely
C. Subgroups (*k* = 2)	bvFTD vs. PD/LBD	385	95	480	–	–	–	0.771	0.718–0.824	–	–	Subgroup
C. Subgroups (*k* = 2)	PPA vs. PD/LBD	385	126	511	–	–	–	0.807	0.757–0.852	–	–	Subgroup
D. Sensitivity analyses	Primary *k* = 2	385	744	1,129	70.6	58.4	12.2	0.785	0.757–0.814	0.922	0.525	
D. Sensitivity analyses	Age-matched (caliper 2 y)	336	336	672	68.7	69.0	−0.3	0.794	0.761–0.826	0.729	0.714	
D. Sensitivity analyses	Age-restricted (55–75 y)	269	445	714	67.2	64.6	2.6	0.777	0.741–0.814	0.84	0.591	
D. Sensitivity analyses	Age-only	385	744	1,129	70.6	58.4	12.2	0.721	0.691–0.751	0.599	0.761	
D. Sensitivity analyses	Slopes + age	385	744	1,129	70.6	58.4	12.2	0.867	0.845–0.888	0.724	0.86	
D. Sensitivity analyses	Phenotyped-FTD-only	385	233	618	70.6	62.8	7.7	0.799	0.763–0.834	0.605	0.836	

AUC, area under the receiver operating characteristic curve; CI, confidence interval; bvFTD, behavioral variant frontotemporal dementia; PPA, primary progressive aphasia; PD/LBD, Parkinson’s disease or Lewy body dementia; FTD, frontotemporal dementia. Sensitivity denotes FTD detection and specificity denotes PD/LBD detection at the Youden-optimal operating point. Age-matched analysis used 1:1 nearest-neighbor matching on baseline age with a 2-year caliper (deterministic greedy, fixed order). Phenotyped FTD was defined by first-visit NACC-derived FTLDSUBT values 1, 2, or 3; all other, unknown, not-assessed, or missing values were excluded from the phenotyped-FTD-only analysis.

### Classification and statistical analysis

We trained random forest classifiers with 300 trees, balanced class weighting, and a fixed random seed (seed = 42) under five-fold stratified cross-validation. Results were stable across random seeds: *k* = 2 out-of-fold AUC was 0.785, 0.786, and 0.787 for seeds 42, 123, and 456, respectively, with maximum absolute deviation 0.003. Performance was summarized as area under the receiver operating characteristic curve (AUC) with 95% confidence intervals from 500 nonparametric bootstrap resamples of out-of-fold predictions. We defined an apparent diminishing-returns threshold heuristically as the smallest k whose confidence interval overlapped with all higher-k configurations. This overlap rule served as a practical screening heuristic rather than a formal equivalence test.

We performed additional analyses. First, a *k* = 2 interval analysis stratified participants by inter-assessment spacing: 6–12 months, 12–18 months, and > 18 months. Second, subgroup analyses compared behavioral variant FTD versus PD/LBD and primary progressive aphasia versus PD/LBD at *k* = 2. Third, a time-to-predicted-probability analysis among participants with at least six visits used a predicted-probability threshold exceeding 0.90, tracking the cumulative proportion reaching this threshold from *k* = 2 through *k* = 6. This exploratory analysis used a separate 500-tree random forest trained on the full *k* = 6 long-follow-up sample; because it was not out-of-fold and probabilities were not calibrated, a predicted probability above 0.90 should not be interpreted as 90% diagnostic certainty. Formal calibration assessment, including reliability curves and Brier scores, was deferred to future prospective work. Fourth, reviewer-requested sensitivity analyses tested age-matched classification using 1:1 nearest-neighbor matching on baseline age with a 2-year caliper (deterministic greedy, fixed order), an age-restricted 55–75 year analysis, an age-only classifier, a slopes-plus-age classifier, and a phenotyped-FTD-only classifier excluding FTD participants without FTLDSUBT values 1, 2, or 3. Sensitivity, specificity, and balanced accuracy were calculated at the Youden-optimal operating point, with sensitivity defined as FTD detection and specificity defined as PD/LBD detection.

Feature importance was extracted from the random forest model at each k value using mean decrease in impurity, permitting visualization of how subdomain contributions shifted with accumulating assessments.

All analyses were conducted in Python 3.10 using scikit-learn 1.3.0. The study used only de-identified, publicly available NACC data and was exempt from additional institutional review board approval per Mayo Clinic policy.

## Results

### Progressive inclusion performance

**FIGURE 1:**
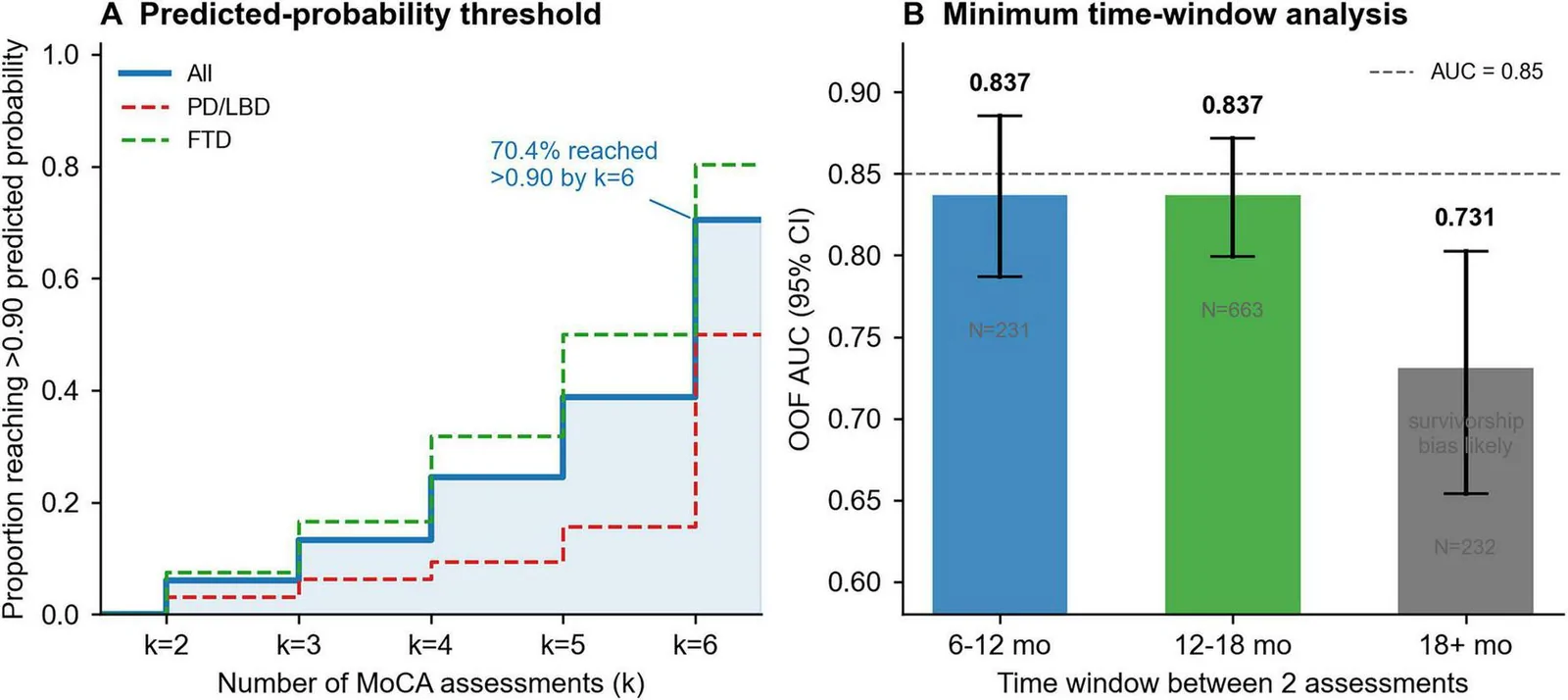
AUC learning curve and feature importance evolution. **(A)** Shows out-of-fold AUC with 95% confidence intervals across *k* = 2 to *k* = 8 serial assessments. The apparent, sample-size-limited plateau occurs at *k* = 4, where confidence intervals overlap with later configurations. **(B)** Shows feature importance by MoCA domain across increasing visit counts; attention dominates early, while language rises later.

At *k* = 2, PD/LBD versus FTD classification achieved AUC = 0.785 (95% CI 0.757–0.814; *n* = 1,129; Figure 1A). At the Youden-optimal operating point, *k* = 2 sensitivity for FTD detection was 0.922, specificity for PD/LBD detection was 0.525, and balanced accuracy was 0.723. This specificity is only marginally above chance and partly reflects the imbalanced 744:385 FTD-to-PD/LBD class structure; in the balanced age-matched cohort, sensitivity and specificity converged to 0.729 and 0.714. Performance increased to 0.812 (95% CI 0.777–0.843) at *k* = 3 (*n* = 756) and 0.831 (95% CI 0.791–0.873) at *k* = 4 (*n* = 391), where sensitivity was 0.923, specificity was 0.673, and balanced accuracy was 0.798. Confidence intervals overlapped with later configurations as the eligible sample contracted from 391 at *k* = 4 to 98 at *k* = 6, indicating an apparent, sample-size-limited plateau rather than proven signal saturation. Full progressive inclusion results are presented in Table 2.

Baseline characteristics for the *k* = 2 analytic cohort are summarized in Table 1. PD/LBD participants were older (mean 70.6 vs. 58.4 years) and less often female (23.6% vs. 47.2%) than FTD participants, while baseline total MoCA scores were similar between groups (22.6 vs. 22.7). The 12.2-year age difference reflects known epidemiological characteristics of these conditions. The primary classifier used only MoCA domain slopes to isolate the trajectory signal and avoid a model that merely re-encoded the known epidemiological age separation; the slopes-plus-age model is therefore presented as a secondary, if-age-available classifier. Completed sensitivity analyses are reported in the sensitivity-analysis rows of Table 2. After 1:1 age matching within a 2-year caliper, the residual age gap was −0.3 years and discrimination remained similar to the primary analysis (AUC = 0.794 (95% CI 0.761–0.826); sensitivity = 0.729; specificity = 0.714). In the age-restricted 55–75 year cohort, AUC was 0.777 (95% CI 0.741–0.814). Age alone yielded AUC = 0.721 (95% CI 0.691–0.751), while slopes plus age yielded AUC = 0.867 (95% CI 0.845–0.888). Within the FTD group, the NACC-derived first-visit FTLDSUBT variable identified behavioral variant FTD (*n* = 95), PPA (*n* = 126), FTD with motor neuron disease (*n* = 12), and other, unknown, not-assessed, or missing FTD subtype labels (*n* = 511). Thus, 233 participants were phenotyped and 511 of 744 FTD participants (68.7%) were unphenotyped by FTLDSUBT. The reviewer-noted approximately 54% unspecified rate reflects a broader phenotyping definition that also counts NACCPPA- or NACCFTDM-flagged participants as subtyped (yielding 416/744, 55.9% unspecified); we report the more conservative single-variable FTLDSUBT definition (511/744, 68.7% unspecified), which does not assume a subtype without an explicit FTLDSUBT value. FTLDSUBT = 2 aggregates all PPA variants; PI review of NACCPPAG found one logopenic-variant PPA case within this group, making AD-pathology contamination through lvPPA negligible in this cohort. Excluding the unspecified FTD group retained 233 phenotyped FTD participants and yielded AUC = 0.799 (95% CI 0.763–0.834).

### Interval analysis

**FIGURE 2:**
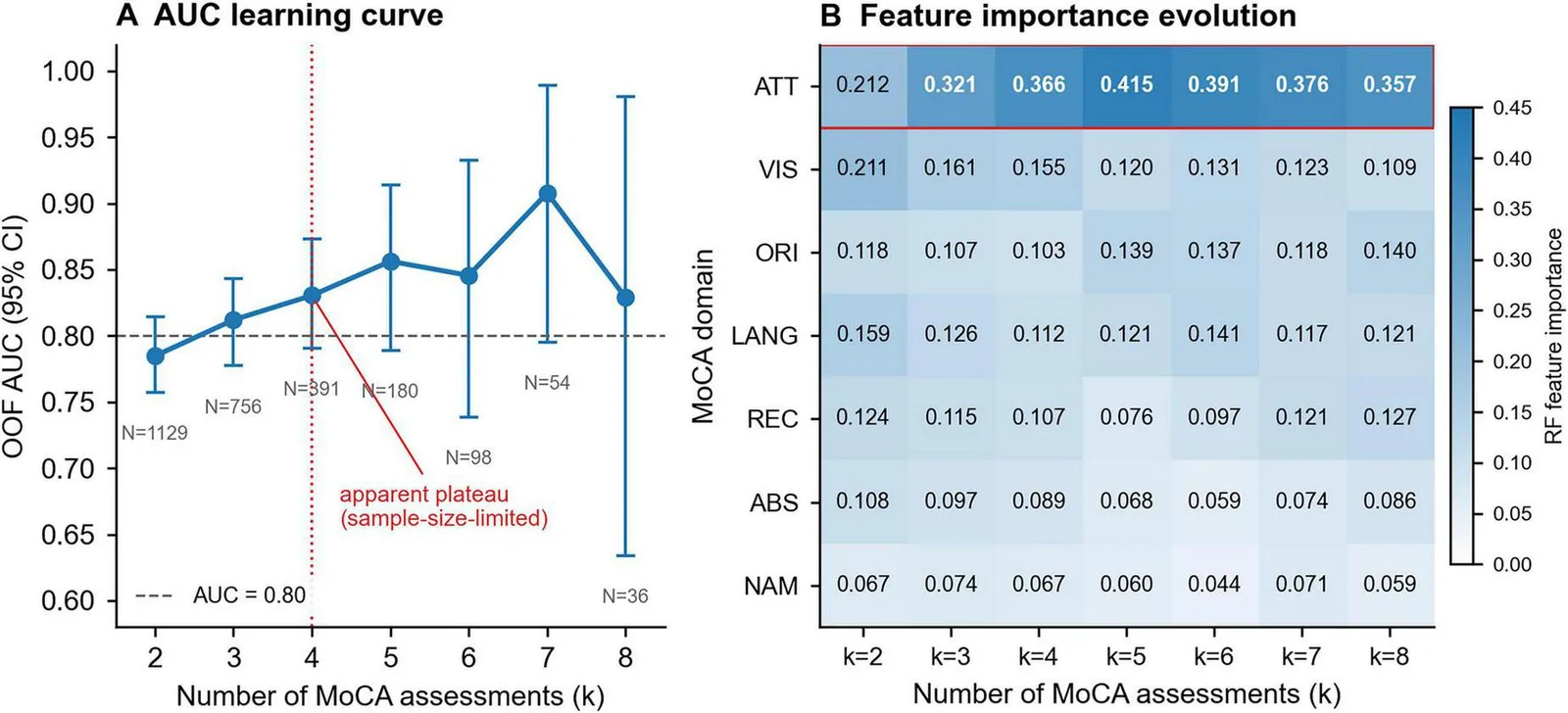
Time-to-predicted-probability threshold and minimum time-window analysis. **(A)** Shows cumulative attainment of predicted probability greater than 0.90 among participants with at least 6 serial assessments; this exploratory analysis used a full-sample 500-tree model and uncalibrated probabilities. **(B)** Shows *k* = 2 AUC stratified by inter-assessment interval, with equivalent performance at 6–12 and 12–18 months and lower performance beyond 18 months.

For the minimum-data scenario at *k* = 2, two assessments separated by 6–12 months yielded AUC = 0.837 (95% CI 0.787–0.885; *n* = 231; Figure 2B), and 12–18 month spacing yielded the same AUC of 0.837 (95% CI 0.800–0.872; *n* = 663). Intervals longer than 18 months showed lower discrimination (AUC = 0.731, 95% CI 0.654–0.803; *n* = 232), likely reflecting survivorship bias and differential dropout. The equivalence of 6–12 and 12–18 month intervals is clinically relevant: it indicates that the triage signal does not require rapid reassessment and can be captured within standard follow-up schedules already in place at most neurology clinics.

### Feature importance dynamics

Attention and visuospatial/executive slopes were co-dominant at *k* = 2 (Figure 1B). Attention became the single dominant feature from *k* = 3 through *k* = 5, while language slope rose steadily with accumulating data and ranked second by *k* = 6. This temporal hierarchy is a hypothesis-generating association rather than mechanistic evidence: mean-decrease-in-impurity feature importance can favor continuous or correlated features and should not be interpreted causally. The late language-importance pattern was also estimated in the same survivorship-biased long-follow-up subset that constrains the higher-k AUC analyses. In addition, the MoCA language subdomain is only a 3-point measure based on sentence repetition and fluency; it does not directly measure the anomia or agrammatism that define PPA, and short-interval change in such a coarse domain can approach test-retest resolution. The attention-plus-visuospatial simplified-triage idea remains untested and would require direct validation before use.

At *k* = 2, subgroup performance remained clinically informative for behavioral variant FTD versus PD/LBD (AUC = 0.771, 95% CI 0.718–0.824) and primary progressive aphasia versus PD/LBD (AUC = 0.807, 95% CI 0.757–0.852).

### Time-to-predicted-probability threshold

Among participants with at least six serial assessments (*n* = 98), 70.4% reached a predicted probability exceeding 0.90 by the sixth visit (Figure 2A). Among the 69 participants who reached the threshold, the median number of assessments required was five; the remaining 29 did not reach it within six visits. Because this exploratory analysis used a full-sample 500-tree model rather than out-of-fold predictions and did not calibrate probabilities, the 70.4% estimate may be optimistic and should not be read as a calibrated confidence statement. The analysis provides a patient-level operational metric for testing-priority workflows: individuals who do not reach the threshold through cognitive trajectory alone represent candidates for whom expedited biomarker confirmation would add the most diagnostic value.

## Discussion

The central finding of this study is that two serial MoCA assessments separated by 6–12 months achieve AUC = 0.837 for PD/LBD versus FTD discrimination in a retrospective cohort. In an indirect cross-study comparison, this point estimate is near the lower edge of fluid biomarker panel reports, while its lower confidence bound (0.787) remains below the 0.85–0.95 range reported for some specialized biomarker studies (Hansson et al., 2022; Siderowf et al., 2023). The comparison should therefore be interpreted as a triage benchmark rather than evidence of biomarker-equivalent accuracy. The MoCA trajectory approach operates at zero marginal cost beyond standard clinical follow-up and requires no invasive sampling, specialized equipment, or reference laboratory access. In settings where biomarker infrastructure is unavailable – which describes much of dementia care globally – serial MoCA trajectory analysis could serve as a first-line prioritization tool, helping determine urgency for confirmatory biomarker evaluation.

The progressive inclusion analysis revealed a practical insight: performance at four assessments was high (AUC = 0.831), but later configurations did not show a stable additional gain. This should be interpreted as an apparent, sample-size-limited plateau. The eligible cohort contracted from 391 participants at *k* = 4 to 98 at *k* = 6, and the *k* = 6 confidence interval widened substantially (95% CI 0.739–0.933), so the observed flattening cannot be distinguished from a true biological saturation point. For clinical implementation, the *k* = 4 result still suggests that a useful triage decision may often be reached within 18–24 months of the first MoCA administration, but the data do not prove that later assessments add no information.

The temporal hierarchy of feature importance offers hypothesis-generating associations about the cognitive signatures linked to classification. Attention slope dominates early discrimination (*k* = 2 through *k* = 5), consistent with the attentional and executive profile described in PD/LBD cohorts and with behavioral and language-predominant FTD clinical phenotypes (Gorno-Tempini et al., 2011; Biundo et al., 2020). Language slope rises in importance with accumulating data, but this should not be read as direct evidence of PPA language biology because the MoCA language subdomain is coarse and the later-k estimates come from a survivorship-biased subset. A staged attention/visuospatial-first workflow is therefore a speculative implementation idea requiring direct model testing, not an established simplified classifier.

The 12.2-year age gap between PD/LBD and FTD groups remains an important limitation, but it was directly tested in the revised analysis rather than deferred. In a 1:1 age-matched cohort using nearest-neighbor matching on baseline age with a 2-year caliper (deterministic greedy, fixed order), mean ages were 68.7 years in PD/LBD and 69.0 years in FTD, leaving a residual gap of −0.3 years; classification performance remained similar to the primary analysis [AUC = 0.794 (95% CI 0.761–0.826)]. This match raised FTD mean age from 58.4 to 69.0 years and discarded 408 FTD participants, so it tests an older, late-onset, atypical FTD subset and should be interpreted as a conservative floor within the age-overlap region rather than a representative-population estimate. The greedy match order was fixed and was not sensitivity-checked across alternative match orders. Restricting the cohort to the 55–75 year overlap band also preserved discrimination [AUC = 0.777 (95% CI 0.741–0.814)]. Age alone separated the groups less well [AUC = 0.721 (95% CI 0.691–0.751)] than MoCA slopes, while adding age to the slope features increased AUC to 0.867 (95% CI 0.845–0.888). These results indicate that age contributes diagnostic information in this cohort, but the MoCA slope signal is not reducible to age alone; the slopes-plus-age model may be useful as a secondary classifier when age is available, whereas the slopes-only primary model isolates trajectory information.

These results support a staged testing-prioritization framework rather than replacement of confirmatory diagnostics. At the first follow-up visit (6–12 months after baseline), the two-visit MoCA trajectory classifier could generate an initial triage probability to help rank urgency for biomarker or specialist confirmation. Patients with high predicted probability would still require clinical confirmation before disease-specific management decisions, while lower-probability or ambiguous cases could be monitored longitudinally and re-prioritized as new MoCA data accrue. This selective referral strategy is intended to organize testing order and urgency, not to license skipping confirmation.

The operating-point profile is also consistent with a testing-prioritization tool, but its limitations should be explicit. In the primary *k* = 2 model, the Youden point favored sensitivity over specificity (0.922 vs. 0.525), leaving PD/LBD specificity only marginally above chance; this asymmetric operating point is partly a consequence of the 744:385 class imbalance and Youden-threshold selection. The high sensitivity is a safer error profile when the clinical priority is to miss fewer FTD cases before confirmatory workup. After age matching, sensitivity and specificity converged to 0.729 and 0.714, respectively, but this operating-point shift should be attributed mainly to class rebalancing to a 50/50 cohort plus re-optimization of the Youden threshold; AUC is rank-based and prevalence-invariant, whereas the selected operating point is not.

Several limitations warrant consideration. First, the NACC cohort represents a convenience sample of participants enrolled at Alzheimer’s Disease Research Centers, which may not reflect the full spectrum of community-presenting neurodegenerative disease. Participants in NACC tend to be more highly educated and more frequently of European ancestry than the general population, potentially limiting generalizability to diverse clinical populations. Second, the PD/LBD reference class could not be reproducibly partitioned into mutually exclusive PD, Parkinson’s disease dementia, and dementia with Lewy bodies subgroups because NACC etiologic flags are overlapping and incomplete; 96 of 385 PD/LBD participants lacked a definite PD or LBD flag, with many values coded not assessed. PD/LBD was therefore analyzed as a combined Lewy-body-spectrum class, and subtype-resolved performance, such as dementia with Lewy bodies versus FTD, could not be estimated in this dataset. Third, FTLDSUBT phenotyping was absent, unknown, not assessed, or otherwise unspecified for 511 of 744 FTD participants (68.7%), making reference-standard heterogeneity a major limitation. Excluding unspecified FTD shifted the operating point from sensitivity/specificity 0.922/0.525 to 0.605/0.836, showing that the unspecified group influences the primary classifier threshold and error profile. Fourth, our sample size at higher k values contracted substantially (*n* = 98 at *k* = 6), widening confidence intervals and limiting the precision of performance estimates beyond the apparent, sample-size-limited plateau. Millar et al. analyzed 156 participants for longitudinal MoCA subtest trajectory modeling in Parkinson’s disease and Lewy body dementia, illustrating that serial cognitive trajectory studies often face sample-size constraints at extended follow-up (Biundo et al., 2020). Fifth, the progressive inclusion design introduces survivorship bias at higher k values: participants completing six or more assessments are more likely to have slower disease progression, higher socioeconomic status, and greater healthcare access than those who drop out earlier. This means the apparent plateau at *k* = 4 may partly reflect reduced sample heterogeneity rather than true signal saturation in MoCA domain slopes. The primary clinical comparison (*k* = 2, interval-stratified) uses the full cohort and is less susceptible to this bias. Additionally, although NACC aggregates data from over 30 Alzheimer’s Disease Research Centers, we did not perform leave-one-site-out validation to assess cross-center generalizability. MoCA administration practices and diagnostic classification thresholds may vary across centers, introducing site-level heterogeneity that could inflate or deflate AUC estimates depending on whether site effects correlate with diagnostic group membership. Because NACC diagnoses are clinically assigned by consensus teams without universal neuropathological confirmation, misclassification in the diagnostic reference standard may be differential across PD/LBD and FTD syndromes, so the direction of bias on AUC is unknown and could either attenuate or inflate observed performance. External validation in an independent cohort and prospective evaluation in a clinical testing-prioritization workflow are essential next steps before implementation. The 6–12 month slope analyses also approach MoCA test-retest resolution for short subdomain scales, and the similar 6–12 versus 12–18 month AUCs suggest that the *k* = 2 signal may partly reflect baseline subdomain-profile differences rather than a fully resolved trajectory. Medication state is another uncontrolled confound: cholinesterase inhibitors and dopaminergic therapy can alter MoCA subdomain performance and therefore the slope signal being classified, but medication status was not available or modeled. Performance was evaluated across approximately 18 configurations, including *k* = 2 through *k* = 8, three interval strata, two subgroups, and six sensitivity models, without formal multiple-comparison correction; the analysis was not pre-registered, so consistency across configurations mitigates but does not eliminate multiplicity concerns.

The clinical implications of these findings extend beyond the specific PD/LBD versus FTD comparison. The biomarker-sparing triage framework we describe could be adapted to other neurodegenerative disease pairs where cognitive trajectory patterns diverge, such as Alzheimer’s disease versus FTD or PD/LBD versus vascular cognitive impairment. Serial MoCA administration is already standard practice in many neurology clinics; the additional step of computing subdomain slopes and applying a trained classifier requires minimal computational infrastructure. We envision a staged testing-prioritization protocol in which serial MoCA trajectory analysis serves as a first-pass screen for ranking confirmatory biomarker or specialist evaluation, with predicted probabilities treated as uncalibrated model outputs rather than a stand-alone diagnosis. This approach could reduce unnecessary delays in biomarker testing, shorten diagnostic timelines, and extend differential diagnostic support to resource-limited settings where advanced biomarker infrastructure is not yet available.

## Data Availability

The original contributions presented in this study are included in the article/supplementary material, further inquiries can be directed to the corresponding author.

## In Memoriam

The late Dr. Sanjeet S. Grewal conceived the clinical question motivating this work and provided neurosurgical perspective on deep brain stimulation patient selection that informed the triage framing. His mentorship shaped the analytical approach.
